# Proteomic Analysis of the Chlorophyta *Dunaliella* New Strain AL-1 Revealed Global Changes of Metabolism during High Carotenoid Production

**DOI:** 10.3390/md15090293

**Published:** 2017-09-20

**Authors:** Faten Ben Amor, Fatma Elleuch, Hajer Ben Hlima, Matthieu Garnier, Bruno Saint-Jean, Mohamed Barkallah, Chantal Pichon, Slim Abdelkafi, Imen Fendri

**Affiliations:** 1 Unité de Biotechnologie des Algues, Biological Engineering Department, National School of Engineers of Sfax, University of Sfax, Sfax 3038, Tunisia; faten_benamor@yahoo.fr (F.B.A.); hajer_benhlima@yahoo.fr (H.B.H); mohamedbarkallah@gmail.com (M.B.); slim.abdelkafi@enis.tn (S.A.); 2 Laboratoire de Biotechnologie Végétale Appliquée à l’Amélioration des Cultures, Faculty of Sciences of Sfax, University of Sfax, Sfax 3000, Tunisia; fatma.elleuch@ymail.com; 3 Center for Molecular Biophysics (CBM), CNRS UPR 4301, Orléans 45071, France; pichon@cnrs-orleans.fr; 4 Laboratoire BRM-PBA Ifremer, Nantes 44311, France; Matthieu.Garnier@ifremer.fr (M.G.); Bruno.Saintjean@ifremer.fr (B.S.-J.)

**Keywords:** proteome, *Dunaliella*, 18S rRNA gene, carotenoids, bidimensional gel electrophoresis, mass spectrometry

## Abstract

The green microalgae *Dunaliella* genus is known for the production of high added value molecules. In this study, strain AL-1 was isolated from the Sebkha of Sidi El Hani (Sousse, Tunisia). This isolate was identified both morphologically and genetically via 18S rRNA gene sequence as a member of the genus *Dunaliella*. Strain AL-1 was found to be closely related to *Dunaliella salina*, *Dunaliella quartolecta* and *Dunaliella polymorpha* with more than 97% similarity. Response surface methodology was used to maximize carotenoid production by strain AL-1 by optimizing its growth conditions. The highest carotenoid content was obtained at salinity: 51, light intensity: 189.89 μmol photons·m^−2^·s^−1^, and nitrogen: 60 mg·L^−1^. Proteomic profiling, using two-dimensional gel electrophoresis, was performed from standard and optimized cultures. We detected 127 protein spots which were significantly differentially expressed between standard and optimized cultures. Among them 16 protein spots were identified with mass spectrometry and grouped into different functional categories using KEGG (Kyoto Encyclopedia of Genes and Genomes) such as photosynthetic Calvin cycle, regulation/defense, energy metabolism, glycolysis, and cellular processes. The current study could be of great interest in providing information on the effect of stressful conditions in microalgae carotenoid production.

## 1. Introduction

Due to a number of benefits, microalgae have recently gained much interest. In fact, they are easy to cultivate and they present high biomass productivity. In addition, their cultivation does not need arable land, they do not vie with food production, and they can produce a great variety of secondary metabolites by adaptation to environmentally changing conditions [1,2]. These molecules have a high potential to be used in biotechnological applications [1].

In the few last years there has been a growing demand for antioxidant, colorant, and bioactive compounds obtained from natural sources [3]. As a matter of fact, carotenoids synthesized by microalgae have been attracting attention. These pigments provide protection to the photosynthetic apparatus in plants by dissipating excess energy [2]. They also take part in oxygenic photosynthesis, as accessory pigments for harvesting light or as structural molecules that stabilize protein folding in the photosynthetic apparatus [4]. Moreover these lipophilic compounds function as scavengers of reactive oxygen species which mainly arise from sunlight absorption by chromophores and consequently protect chlorophylls, proteins, lipids, and DNA from oxidative damages. Carotenoids produced by green algae, are attractive materials in food, pharmaceutical, and cosmeceutical industries due to their strong properties. They have been associated in recent years with lowering the risks of cancer [5].

The aptitude to produce these large amounts of specific organic compounds by some *Dunaliella* members has made them superlative organisms for cell factory applications [6]. Amongst others, *Dunaliella salina* (*Chlorophyta*, *Chlorophyceae*), a unicellular biflagellate green alga, has been highly researched and utilized as a source of various industrial compounds such as carotenoids, glycerol, and unsaturated fatty acids [7].

The unicellular green algae *D*. *salina* can be considered as a powerful biological system able to tolerate and acclimate to a wide range of salt concentrations by production and accretion of the compatible solute glycerol at concentrations that are relative to the external NaCl [8,9]. Additionally to their halotolerance; some *Dunaliella* species display remarkable properties of tolerance to high light [10,11]; low temperature, and limited nutrients [12], achieved by the buildup of massive amounts of carotenoids, glycerol, unsaturated fatty acids, and many other compounds.

The halotolerant algae *D*. *salina* acclimate to various kinds of environmental stress by regulating the expression of numerous stress inducible genes [13]. This is achieved by some changes in metabolism involving the up-regulation or down-regulation of many metabolic pathways.

Rapidly developing genomics, transcriptomics, proteomics, and metabolomics have become crucial in understanding the microorganism’s response mechanism to the changes occurring in their physical environment [14]. Proteomic study and two-dimensional electrophoresis (2-DE), have been the main techniques of separation and comparison of complex protein mixtures in *D. salina* [15]. When consolidated with mass spectrometry [16], these combined methods could precisely determine molecular mass and analyze the molecular structure [17,18].

In this paper, a response surface methodology (RSM) was applied to determine the influence of culture conditions (light intensity, salinity, and nitrogen deficiency) on carotenoid production. We analyzed the protein expression during carotenoid accumulation in the isolated strain AL-1 with 2-DE. A differential protein expression map, mass spectrometry, and bioinformatics analysis were used to analyze and identify the differentially expressed proteins with the aim of extending our knowledge on physiological changes in cells under stressful conditions.

## 2. Results and Discussion

### 2.1. Identification of the Newly Isolated Dunaliella Strain

To valorize strains belonging to the Tunisian biotope, which have bioactive compounds that can be used in biotechnological applications, a new halotolerant microalga was isolated from Sebkha of Sidi El Hani (Sousse, Tunisia). The cell shape of our isolate varies from cylindrical, ovoid, and fusiform to about spherical. This strain is unicellular and biflagellate. The cellular size is measured as approximately 8–18 μm diameter. These characteristics are consistent with previous morphological characteristics of *Dunaliella* genus [19]. The 18S rRNA gene sequence of strain AL-1 (comprising about 1800 bp) was amplified using the universal primers EukA and EukB as described by Chtourou et al. [20,21] to analyze the strain phylogenetic position. Phylogenetic analysis showed that strain AL-1 is a member of *Dunaliellaceae* family and *Dunaliella* genus. The isolated strain was found to be closely affiliated to *D*. *salina*, *D*. *quartolecta,* and *D*. *polymorpha* with more than 97% similarity. 

Microalgae cells change shape (fusiform to spherical) and color (green to orange) with changing cultural conditions (under optimized conditions).

### 2.2. Experimental Design and Validation of Optimized Growth Conditions

Previous findings have shown that within stressful conditions such as high light intensity [10], salt concentration [9] or nitrogen deficiency [12], *Dunaliella* strains could accumulate a high amount of carotenoids.

In order to ameliorate the carotenoid production of our isolated strain, an optimization of the culture conditions was performed considering preliminary analyses of nitrogen concentration (NaNO_3_), salinity (NaCl), and light intensity (Table 1).

**Figure 1:**
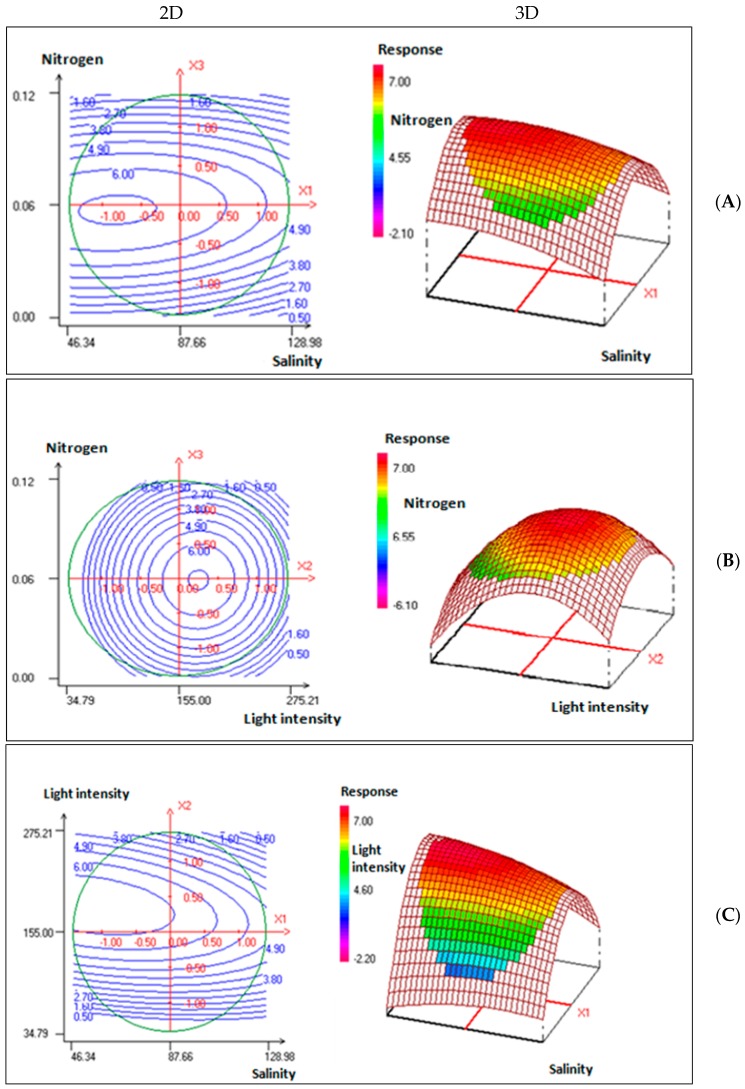
(**A**) Contour plots and response surface plot showing the effect of nitrogen, salinity concentration, and their mutual interaction on fixed light intensity. (**B**) Contour plots and response surface plot showing the effect of nitrogen concentration, light intensity, and their mutual interaction on salinity concentration fixed. (**C**) Contour plots and response surface plot showing the effect of light intensity, salinity concentration, and their mutual interaction on nitrogen concentration fixed.

A response surface methodology (RSM) was then used to examine the effect of interactions between these factors on carotenoid production. Nemrod-W 2D and 3D plots (Figure 1; Table 2) show that the established model indicates that only b_2_ is slightly significant while the quadratic effects b_22_ and b_33_ have a very significant effect on the production of microalga carotenoids.

In fact, the carotenoid production from cultures performed at optimized conditions was three times higher than that from cultures in F/2 medium under standard conditions. It increased effectively from 1.98 mg·g^−1^, mass spectrometry (MS) at light intensity of 80 μmol photons·m^−2^·s^−1^, 27 salinity and 1.075 g·L^−1^ nitrogen to 6.86 mg∙g^−1^ MS at light intensity 189.89 μmol photons·m^−2^·s^−1^, 51.01 salinity and 60 mg·L^−1^ nitrogen. This result confirms the successful application of the Box-Behnken methodology to optimize factors and study their interaction for the best carotenoid production.

The variance’s analysis (ANOVA) through Fisher’s *F* test of the carotenoid production of *Dunaliella* sp. was performed to ensure the statistical significance and the adequacy of the model. The model was found to be significant at 95% confidence level by the *F*-test as shown in Table 3, with a *p*-value of regression ≤0.05. In addition, the model did not exhibit lack of-fit (*p* >0.05) denoting the failure of the model to represent data in the experimental domain at points that were excluded from the regression [22].

The obtained model quality was evaluated by calculating of the correlation coefficient value as well as the determination coefficient (*R*^2^).

The experimental results were analyzed and an approximating function of *Dunaliella* sp. carotenoid production was obtained in the form of the Equation (1):

(1) $$\begin{array}{l} Y_{\mathrm{carotenoids}}=6.421-0.513X_{1}+1.395X_{2}-0.125X_{3}-0.327X_{1}^{2}-2.781X_{2}^{2}-2.305X_{3}^{2}-0.646X_{1}X_{2}\\ +0.188X_{1}X_{3}+0.135X_{2}X_{3} \end{array},$$

where *Y*_carotenoids_ represent the predicted carotenoid content; *X*_1_ represent the salinity; *X*_2_ the light intensity, and *X*_3_ the nitrogen.

As a result, the optimized conditions for carotenoid yield were as follows: salinity, 51.01; light intensity, 189.89 μmol photons·m^−2^·s^−1^, and nitrogen, 60 mg∙L^−1^.

The *R*^2^ value of 0.924 for Equation (1) was relatively high and indicated that the experimental carotenoid content values are in good agreement with the predicted carotenoid production levels from this model. The interaction between each independent factor and *R*^2^ can be considered statistically significant.

### 2.3. Proteomic Analysis

#### 2.3.1. Two-Dimensional Electrophoresis Profiles of Total Proteins in Response to Carotenoid High Production

**Figure 2:**
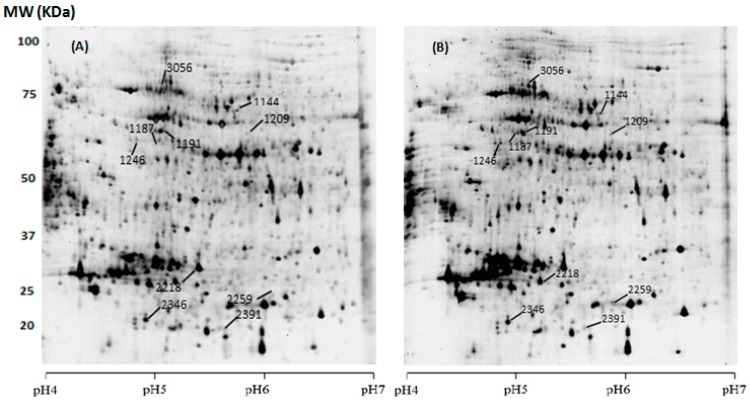
2-DE of whole cell proteoms of *Dunaliella* AL-1 strain at exponential phase of standard (**A**) and optimized (**B**) cultures. Proteins of both cultures at exponential phase were extracted. For each technical duplicates, 30 μg of whole cell proteins were separated on a pH 4–7 gradient and 12% polyacrylamide sodium dodecyl sulfate (SDS) gel and then revealed by silver staining. Six gels were included for image and statistical analysis. Identified spots by mass spectrometry (MS) are localized on the gels.

In order to extend our knowledge of the molecular mechanisms implicated in the production of carotenoids under stress conditions, we undertook a differential comparative proteomic analysis of the isolated strain growing in standard and optimized cultures for production of these pigments. As shown in (Figure 2) and based on SameSpot software analysis, the spots revealed significant difference in proteome from the newly isolated strain cultured in two metabolic conditions (standard and optimized). Our proteomic study resulted in analysing 1173 protein spots of which 127 were differentially expressed. Among them, 57 protein spots were down-regulated and 70 were up-regulated.

The present work showed that exposure to these stressful environmental conditions distorted the accumulation of many *Dunaliella* strain AL-1 proteins. These proteins were found to be involved in a different metabolic pathway positioning through KEGG (Kyoto Encyclopedia of Genes and Genomes), whose functions were involved in carbon and energy metabolism, carbohydrate metabolism, glycolysis/gluconeogenesis, protein chaperone, and cellular processes.

#### 2.3.2. Spot Identification

Protein spots that showed consistent differences in all gel images and detected by SameSpot Software (Figure 2) were selected for automated MS/MS spectrometry analysis and peptide mass fingerprinting (Table 4 and Table 5). Table 4 also shows if the particular spot was up- or down-regulated under optimized conditions, promoting high carotenoid production. We found that 11 spots contained 16 unique proteins (Table 5). Some of these proteins, like heat shock protein (HSP), the α- and β-subunit of mitochondrial ATP synthase, enolase, carbonic anhydrase, α- and β-tubulin, were up-regulated in optimized conditions; whereas photosystem I light-harvesting cholrophyll-a/b protein and chloroplast ribulose phosphate-3-epimerase were down-regulated.

#### 2.3.3. Functional Classification of Identified Proteins

The optimized culture conditions for carotenoid production appear to affect considerably the metabolism of *Dunaliella* sp. AL-1. In fact, the potential roles of the differentially expressed proteins associated with applied stress in microalga are examined below.

*ATP synthase CF1 alpha and beta subunits* (chloroplast) (migrated in different spots: 1209, 2218, 1187, 1246, and 1191): The enzyme related to energy metabolism, photosynthesis, and also metabolic flexibility. The great quantity of these enzymes is consistent with proteomics findings demonstrating up-regulation of energy metabolism in the *Dunaliella* strain at high salinity [23]. As described by Liska et al. [23], the increased level of ATP and redox energy generating enzymes suggest enhanced energy metabolism at high salt in *Dunaliella salina*. In accordance with these results, we found that carotenoid production was stimulated 346% at high salinity, in fact, it increased from 1.98 mg·g^−1^ MS under standard conditions (salinity 27) to 6.86 mg·g^−1^ MS under optimized conditions (salinity 51) (Figure 1). Producing more ATP and redox energy could play a role in supplying the metabolic pathways for an enhanced transport of CO_2_ and ions [16].

*Carbonic anhydrase* (spot 3056): Carbonic anhydrase, as a metalloenzyme, is found in almost every type of tissue and plays a crucial role in catalyzing the equilibration of carbon dioxide (CO2) and carbonic acid (H_2_CO_3_) [24]. It is vital for many eukaryotic physiological processes like respiration, photosynthesis, and CO_2_ transport. The activity regulation of carbonic anhydrase is associated with numerous factors and does not follow a similar pattern for all the studied microalgae [25]. Under high carotenoid production conditions, when the salinity reaches 51, the CO_2_ solubility decreases and it becomes the rate-limiting phase in photosynthesis [26]. Our analysis reveals that the carbonic anhydrase was up-regulated in AL-1*Dunaliella* strain. This suggests that carbonic anhydrase enhances carbon acquisition [16]. It should be noted that, previous proteomics analysis showed also an up-regulation of this enzyme with light intensity in *Chlamydomonas reinhardtii* [26]. Similar to the present finding, Dionisio-sese et al. [27] indicated that light caused an increase in cabII-1 transcript abundance in *Chlamydomonas* by virtue of its role in the light reactions of photosynthesis.

*Heat shock protein HSP 70* (spots 1246 and 1256): HSPs are a group of functionally related proteins triggered by various stressful conditions. They have multiple roles including membrane translocation, protein degradation, protein folding, misfolded proteins repair, and protein homeostasis regulation in normal and stressed cells [28]. Low temperature, osmotic, salinity, oxidative, desiccation, heavy metals, UV light, nitrogen deficiency or water deprivation are among the stressful conditions for cells, which have been found to provoke the synthesis of HSPs according to several studies [29]. Therefore, up-regulation of HSP in *Dunaliella* can also be described altogether as part of the stress response [30]. High level expression of HSPs can be generated by exposure to different environmental stress conditions [31]. We identified in this context of stressful conditions (N deficiency, high salinity, high light intensity), two HSPs 70 kDa proteins, which were up-regulated (2.9 fold).

Several studies have shown that nitrogen starvation could induce oxidative stress in plants, which could be part of the production of stress responsive proteins. Hockin et al. found that a lower protein content associated with nitrogen starvation could limit electron transfer over the photosynthetic complex in diatoms, rising the production of ROS, and thus oxidative stress [32]. Recently, some other studies have reported increase in HSP70 expression in response to salinity stress [33,34]. Yokthongwattana et al. [6] studied the involvement of HSP70B in the repair of photodamaged Photosystem II (PSII) and showed the strong correlation between patterns of HSP70B gene expression and the rate of PSII photodamage further with a low light vs high light.

*Enolase* (Spot 1144): The conversion of 2-phosphoglycerate to phosphoenolpyruvate (PEP) is catalyzed by the key enzyme in the glycolytic pathway; the enolase [35]. This abundant glycolytic enzyme is absolutely a multifunctional protein and could be considered as a surface receptor and also as a heat shock protein [36]. As observed, our analysis found up-regulation of the enolase under carotenoid accumulating conditions (3.1 fold). It was noted also that enolase functions as a cell associated stress protein implicated in cellular protection during hypoxia [37]. Recently, Ruan et al. reported that enolase is involved in both temperature and salt stress in algae [38].

*Chloroplast ribulose phosphate-3-epimerase* (Spot 2259): Ribulose phosphate-3-epimerase is involved in the biological process and takes part in several important metabolic pathways, comprising carbohydrate biosynthesis and the Calvin cycle. This enzyme which catalyzes the reversible epimerization of d-ribulose 5-phosphate to d-xylulose 5-phosphate, showed a decrease in expression under carotenoid biosynthesis conditions (5.4 fold). On the contrary, authors have found an increase in the expression level of this protein subsequently with nitrogen deprivation in the diatom *Phaeodactylum tricornutum* [39]. As suggested previously by Nowitzki et al. [40] and Mastrobuoni et al. [41], the increased expression of this enzyme may participate in the increased yield of ribulose-1,5-bisphosphate, hence favoring carbon fixation and production of glyceraldehyde-3-P (G3P), which is the precursor for triglyceride biosynthesis.

*Photosystem I light-harvesting chlorophyll-a/b protein 2* (Spots 2346 and 2391): Protein complexes are major components of the photosynthetic apparatus that absorb light and transfer excitation energy to the photochemical reaction [42]. Previous observations have also mentioned that the functional Chlorophyll antenna size of photosystem I (PSI) was diminished significantly in response to irradiance stress during cell growth [43]. It is concluded that excessive irradiance during cell growth induces a down-regulation of the Chlorophyll antenna size of PSI in *Dunaliella* AL-1.

*Flagellar* *Proteins* (Spots 1187 and 1191): In eukaryotic cells, microtubules are highly dynamic structures composed of, α- and β-tubulin heterodimers. They are known to play a key role in major processes such as cell motility, intracellular trafficking, and mitosis [44,45].

Recent studies have suggested that microtubules plays a major role in plant development as potent players in detecting stressful conditions, and as a consequent cellular response inducer [46].

Our study showed that α- and β-tubulins, known to compose the flagellum, were up-regulated in *Dunaliella* strain AL-1. These findings are in agreements with previous results [23] which explained the functional meaning of increased yield of cytoskeletal proteins under high salt conditions by strengthening the cell cytoskeleton. However, recent results [42] reported a down-regulation of flagellar proteins under salt stress. This metabolism is not yet well known in microalgae, but we suspect that up-regulation of tubulins in response to these stressful conditions may result in a profound reorganization of these membrane skeleton structures involved in plasma membrane integrity as well as harbouring signalling complexes and ion channels. In fact, the response of cytoskeleton protein to stress is well documented in plants. Authors have linked the changes in abundance of cytoskeleton-associated proteins to an adaptive response during osmotic stress [47]. Livanos et al., discussed well the interplay between ROS and tubulin cytoskeleton and highlighted the reorganization and remodelling of tubulin in response to ROS homeostasis [46].

**Figure 3:**
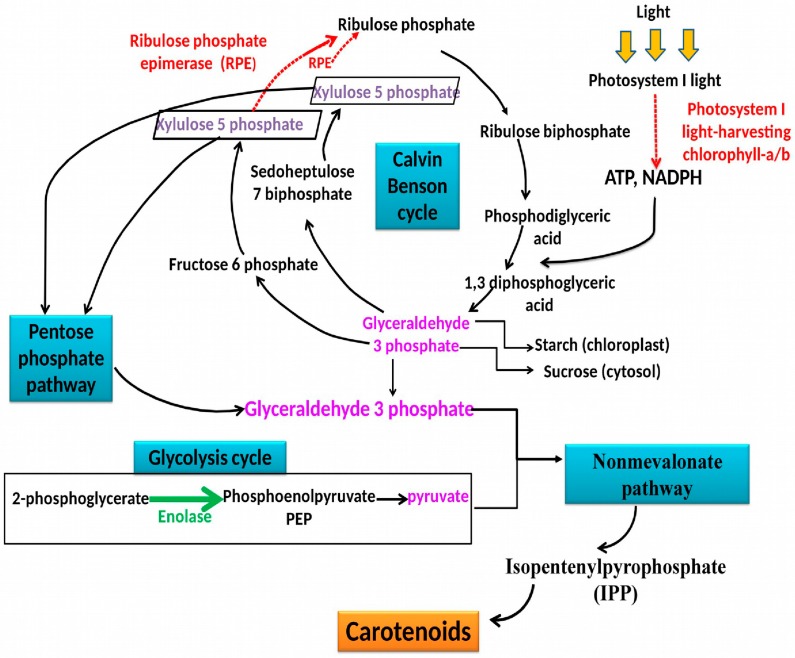
Schematic presentation of the regulated metabolic pathways under carotenoid production conditions in *Dunaliella* strain AL-1. Red dotted arrows indicate the down regulated enzymes while green thick arrows show the up regulated enzyme identified in this work. Blue boxes denote pathway names.

Taken together, the identified carotenoid production induced proteins in *Dunaliella* sp. AL-1 revealed a down-regulation of PSI light proteins and ribulose phosphate-3-epimerase, an important enzyme in the Calvin cycle. These findings show that *Dunaliella* sp. strain AL-1 when producing a high amount of carotenoids, have a reduced photosynthetic CO_2_ assimilation as a consequence. Nonetheless, carbon continued to be assimilated as seen from the up-regulation of carbonic anhydrase. The latter is involved in the first step of CO_2_ fixation in the photorespiration process. Slowing down the light depended-photosynthesis would be offset not only by enhancing photorespiration but also by stimulating carbohydrate degradation (e.g., oxidative pentose phosphate cycle, and glycolysis cycle). In fact, as shown in (Figure 3), the down-regulation of the Calvin cycle enzyme ribulose phosphate-3-epimerase leads to the accumulation of xylulose-5P, a substrate for G3P production in the pentose phosphate cycle. Consistent with this prediction, we found that enolase, an important enzyme in the glycolysis cycle, was also up regulated under carotenoid production conditions. The accumulation of PEP molecules produced by enolase leads to an increased yield of pyruvate, the latest product of glycolysis pathway. The regulated metabolic pathways for a subsequent accumulation of G3P and pyruvate would be probably the first step of the carotenoid production by the methylerythritolphosphate (MEP) pathway.

The present data suggest that carotenoid production conditions are considered as stressful conditions for *Dunaliella* sp. strain AL-1, which in turn reacts by reducing photosynthetic assimilation and accelerating photorespiration. The findings revealed also an important accumulation of the xylulose-5P product. This is described for the first time, to the best of our knowledge, in carotenoid production conditions. It is suspected to play a crucial role as precursor or activator for isopentenyl pyrophosphate (IPP) production in non-mevalonate pathway (Figure 3).

## 3. Materials and Methods

### 3.1. Algal Strain, Medium and Culture Conditions

The alga used in this study was isolated from the Sebkha of Sidi El Hani (Sousse, Tunisia), which is located thirty kilometers from the south-west of Sousse. First, two hundred milliliters of each sample were filtered using a 0.22 μm pore size membrane. Subsequently each membrane was flooded into 50 mL of F/2 medium [48]. Flasks were incubated at 28 °C at a continuous illumination intensity (80 μmol photons·m^−2^·s^−1^) provided by cool-white fluorescence tubes (TL5 tungsten filament lamps; Philips Co., Taipei, Taiwan). Every two days, the cultures were visually examined and the algal development was confirmed by inverted microscopy analysis at 40× magnification (Motic microscope AE2000, Barcelona, Spain) [49].

Isolation of a pure microalga was performed by combining serial dilutions, plating, and micromanipulation techniques. In order to check the culture purity, each dilution series was microscopically analyzed.

### 3.2. DNA Extraction, PCR Amplification, Sequencing, and Phylogenetic Analysis

The microalgal strain was identified based on 18S rRNA gene sequence. Genomic DNA extraction was performed using Quick-G DNA MiniPrep D3006 Kit (Zymo Research, Irvine, CA, USA) as recommended by the manufacturer. The 18S rRNA gene was amplified by PCR using universal primers: *EukA* (5′-AACCTGGTTGATCCTGCCAGT-3′) and *EukB* (5′-TGATCCTTCTGCAGGTTCACCTAC-3′) [21]. PCR condition was as follows: denaturation by heating at 95 °C for 3 min and subjected to 35 cycles at 95 °C for 30 s, at 55 °C for 30 s, and at 72 °C for 2 min and a final elongation step at 72 °C for 7 min [50]. PCR products were purified and sequenced with *EukA* and *EukB* primers. All 18S DNA sequences of the isolated strains were compared to all microalgal sequences available in the Gene Bank data base using the BLAST program.

### 3.3. Carotenoid Analysis 

To quantify carotenoids, 2 mL of each culture in their exponential phase (standard and optimized conditions) were centrifuged at 4000× *g* during 10 min. The pellet was solubilized in ethanol 96% and sonicated for 30 min to extract all pigments. After sonication, the solution was centrifuged at 10,000× *g* for 10 min and carotenoids were assayed spectrophotometrically using the Equation (2) given by Wellburn and Lichtenthaler [51].

[carotenoids] (mg·L^−1^) = (1000 × A_470_ − 2.05 × [chlorophyll a] − 114.8 [chlorophyll b])/245 (2)

### 3.4. Experimental Design and Data Analysis 

In this present paper, a Box-Behnken design [52] was applied to optimize the best experimental conditions of three independent factors affecting the carotenoid production of *Dunaliella* sp.: salinity (*X*_1_), light intensity (*X*_2_), and nitrogen (*X*_3_). The amount of carotenoids (*Y*) was fixed as the design experiments response which could be described by a second order polynomial function. Nemrod-W^®^ software (LPRAI, Marseille, France) was used, for the experimental design, regression and statistical analysis [49,53] (Logiciel Nemrod-W, LPRAI, Marseille, France). Table 1 and Table 2 show independents variables of the Box–Behnken plan in coded levels where *Y* is the response variable, β_0_ the constant, *X*i and *X*j the coded variables ranging between +1 and −1. The coefficients for the linear, quadratic, and interaction effects are. βi, βii and βij, respectively. Furthermore, the experiments were randomized to avoid systematic error, and three central replicates were added to estimate the pure experimental error. In the current work, the experiment design contained 15 trials for optimizing culture conditions and the levels of independent variables were set to three levels: low (−1), medium (0), and high (+1).

### 3.5. Statistical Analysis

Analysis of variance (ANOVA) was used to validate the results of carotenoid production of the RSM design.

### 3.6. Proteomic Analysis

#### 3.6.1. Protein Extraction

For proteomics, 400 mL of exponential phase cultures (standard and optimized) were centrifuged at 2500× *g* for 20 min at 5 °C. Pellets were rinsed twice with distilled water, rapidly frozen at −80 °C then lyophilized. The lyophilized pellets were used for total protein extraction following the protocol reported by Lee and Lo [54]. In brief, cells were added by 1 mL TRIzol Reagent™ (Ambion, Invitrogen, Carlsbad, CA, USA) and protease inhibitors (Roche Diagnostics, Germany) then sonicated for 3 min using a Vibra-Cell^®^ 75022 sonicator (Bioblock, Illkirch, France) in an ice bath. After that 200 μL of chloroform were added to the cell lysate before shaking and centrifugation for 10 min at 4 °C (12,000× *g*). Ethanol (300 μL) was added to the lower phase after removing the hydrophilic one. After centrifugation for 10 min at 4 °C (16,000× *g*), the supernatants was mixed with one volume of 20% trichloroacetic acid (TCA) and 0.14% *β*-mercaptoethanol, in cold acetone. After an overnight incubation, proteins were precipitated at −20 °C, the mixtures were centrifuged for 10 min at 4 °C (16,000× *g*). Finally, the pellets were washed with cold acetone then re-suspended in a buffer containing urea (6 M), thio-urea (2 M), CHAPS (4%), and Bio-Lyte 3/10 (2%) [55]. Two biological replicates were performed for each condition.

#### 3.6.2. Two-Dimensional Gel Electrophoresis (2-DE)

Extracts were analyzed on analytic 2-DE gels by referring to the O’Farrell protocol [56]. The second-dimension electrophoresis was performed on 12% sodium dodecyl sulfate (SDS) polyacrylamide gels to separate proteins having molecular weight ranging from 10 to 120 kDa. 2D Clean-up kit (GE Healthcare, Freiburg, Germany) was used to purify 30 μg and 300 μg of protein for analytic and preparative gels, respectively. The aliquots were then re-suspended in 330 μL rehydration buffer containing urea (6 M), thio-urea (2 M), CHAPS (4%), Bio-Lyte 3/10 (2%), bromophenol blue (0.01%), tributylphosphine (3.3 mM), and DTT (5%). The dry immobilized pH gradient (IPG) strips (linear pH 4–7 (Bio-Rad, Marnes-la-Coquette, France)), were rehydrated for 18 h at 50 V before applying isoelectric focusing (IEF) using the Bio-Rad Protean IEF Cell at 66,000 V·h. The strips were first treated with a buffer containing urea (6 M), SDS (2%), Tris-HCl (0.05 M) pH 8.8, glycerol (30%) added with DTT (2%) and tributylphosphine (3.3 mM). Then they were treated with the same buffer supplemented this time with iodoacetamide (4%) [55]. Finally, the visualization of proteins was carried out by the silver staining and Bio-Safe colloidal Coomassie blue (Bio-Rad, Marnes-la-Coquette, France) methods for analytic and preparative gels, respectively. Two technical replicates were performed for each extract.

#### 3.6.3. Image Acquisition and Data Analysis

Bio-Rad GS800 densitometer was used to register analytic gels images. The latter were analyzed with the Progenesis SameSpots, version 3.0, software (Nonlinear Dynamics Ltd., Newcastle, UK). The quality control (QC) of the software, was used to verify the gel’s quality. The vector alignment tool of SameSpots Workflow was employed for an automatic pixel level geometric alignment of the gels monitored by manual corrections. The background-corrected abundance of each spot was calculated, and the abundance ratio was determined by dividing the sample abundance by the reference abundance. Spot volumes were normalized to calibrate data between different sample runs, and normalized spots were then statistically analyzed by using the statistics module in SameSpots. ANOVA tests were carried out to evaluate significant differences between standard and optimized cultures. The significant differentially expressed protein spots were elaborated using the criterion a *p*-value <0.05 to exclude false positives, a power >0.8 to guarantee reproducibility among gels of standard and optimised cultures, and a fold number >2 for the biological significance.

#### 3.6.4. In-Gel Tryptic Digestion and Protein Identification by Mass Spectrometry

The differentially expressed protein spots of interest were further identified by mass spectrometry (MS). Protein samples of each condition group were run in parallel on separate preparative polyacrylamide gels and stained with Coomassie brilliant blue (Colloidal Blue stain kit; Bio-Rad, Marnes-la-Coquette, France). Bands of interest were excised from gels and automated tryptic digestion was conducted as previously described [57,58]. Gel bands were excised in a sterile laminar flow hood, transferred individually to 1.5 mL microtubes and cut into cubes of roughly 1 mm^3^. Gel cubes were de-stained for 1 h and 30 min at 4 °C using a solution of 45% acetonitrile and 55 mM ammonium bicarbonate. After gel cube washing and in-gel trypsin proteolysis of proteins, the peptides produced were extracted onto Poros beads and purified with ZipTips (Millipore, Molsheim, France) as previously described [58].

Extracted proteolytic peptides were analyzed by nanoUltraHPLC-nanoESI UHR-QTOF MS. Experiments were performed using an UltiMate™ 3000 NanoRSLC System (Dionex, Sunnyvale, CA, USA) connected to a Bruker MaXis UHR-QTOF 2 GHz mass spectrometer equipped with an online nano-ESI ion source. The LC-MS setup was controlled by Bruker Hystar™ software version 3.2 (Bruker Daltonics, Bremen, Germany). Peptides were pre-concentrated online on a Dionex Acclaim PepMap100 C18 reverse-phase precolumn (inner diameter 100 μm, length 2 cm, particle size 5 μm, pore size 100 Å), and separated on a nanoscale Acclaim Pepmap100 C18 column (inner diameter 75 μm, length 25 cm, particle size 2 μm, pore size 100 Å) at a flowrate of 450 nL/min using a 2–35% gradient of acetonitrile in 0.1% formic acid. Peaks with the three highest intensities and a minimum of 400 ion counts were selected for collision-induced dissociation (CID) MS/MS fragmentation using an isolation window of 3–9 Da depending on the *m*/*z* value.

Acquired MS/MS spectra were searched against the UniProtKB/Swiss-Prot/TrEMBL and non-redundant protein sequences from NCBI (http://www.ncbi.nlm.nih.gov) using the Mascot identification engine (version 2.3, Matrix Science, London, UK). The search was conducted allowing for a maximum of two missed cleavages, 5 ppm tolerance for precursor ions, and 0.04 Da for fragment ions, respectively. Methionine oxidation was allowed. Since contaminations from human (mainly keratins) origin could be present in the samples analyzed, the search in databases was restricted to microalgae species. In case of peptides matching to multiple members of a protein family, the presented protein was selected based on both the highest score and the highest number of matching peptides.

## 4. Conclusions

The *Dunaliella* strain AL-1 was isolated and identified based on 18S rRNA gene sequence. Response surface methodology was used to determine the optimal experimental conditions (light intensity, salinity, and nitrogen deficiency) for high carotenoid production from the newly isolated *Dunaliella* AL-1 strain. In this present paper, two-dimensional gel electrophoresis (2-DE) coupled with MS-MS resulted in the understanding of some physiological changes in the cells. In fact, a larger number of different metabolic, stress, and cellular process genes were differentially-expressed under optimized conditions. This allowed us to better understand the metabolic changes in *Dunaliella* sp. strain AL-1 during the production of carotenoids.

## Figures and Tables

**Table 1:** Variables and experimental levels for optimizing culture conditions.

Factor	Coded Symbole	Level		
		−1	0	+1
Salinity	X_1_	58.44	87.66	116.88
Light intensity (μmol photons·m^−2^·s^−1^)	X_2_	70	155	240
Nitrogen·(g·L^−1^)	X_3_	0.02	0.06	0.1

**Table 2:** Statistical analysis of the coefficients for carotenoid response.

Coefficient	Value	Student’s *t* Test	Significance (%)
b_0_	6.421	10.77	0.0120 ^a^
b_1_	−0.513	−1.41	21.9
b_2_	1.395	3.82	1.24 ^c^
b_3_	−0.125	−0.34	74.6
b_11_	−0.327	−0.61	56.9
b_22_	−2.781	−5.17	0.355 ^b^
b_33_	−2.305	−4.29	0.781 ^b^
b_12_	−0.646	−1.25	26.6
b_13_	0.188	0.36	73.1
b_23_	0.135	0.26	80.5

^a^ Significant at 99.9%, ^b^ Significant at 99%, ^c^ Significant at 95%.

**Table 3:** Variance analysis for carotenoid production response.

Source of Variation	Sum of Squares	Degrees of Freedom	Mean Square	Ratio	Significance (%)	Significance
Regression	64.4768	9	7.1641	6.7147	2.47 ^c^	Significant
Residual	5.3346	5	1.0669	-	-	-
Lack of fit	5.1483	3	1.7161	18.423	5.2	Not significant
Error	0.1863	2	0.0932	-	-	-
Total	69.811	14	-	-	-	-

*R*^2^ = 0.924; ^c^ Significant at 95%.

**Table 4:** Identification of *Dunaliella* strain AL-1 proteins induced by carotenoid production conditions.

Spot Number	Hypothetical Function	ANOVA (*p*)	Fold	Standard Conditions	Stressful Conditions	Up/Down
1144	Enolase	3.218 × 10^−4^	3.1	2.936 × 10^5^	8.967 × 10^5^	up
3056	Carbonic anhydrase	5.285 × 10^−4^	3.1	1.958 × 10^6^	6.082 × 10^6^	up
1246	ATP synthase CF1 *beta* subunit (chloroplast)	0.001	4.1	3.382 × 10^5^	1.370 × 10^6^	up
1209	ATP synthase CF1 *alpha* subunit (chloroplast)	0.002	3.4	1.002 × 10^5^	3.448 × 10^5^	up
1187	*beta*-tubulin, partial	0.003	3.1	2.854 × 10^5^	8.967 × 10^5^	up
2391	Photosystem I light-harvesting chlorophyll-a/b protein	0.005	3.5	4.321 × 10^6^	1.227 × 10^6^	down
1191	ATP synthase CF1 *beta* subunit (chloroplast)	0.011	5.0	4.455 × 10^5^	2.232 × 10^6^	up
2259	Chloroplast ribulose phosphate -3-epimerase	0.014	5.4	2.089 × 10^6^	3.860 × 10^5^	down
2218	ATP synthase CF1 *alpha* subunit (chloroplast)	0.021	2.7	3.416 × 10^6^	1.273 × 10^6^	up
2346	Photosystem I light-harvesting chlorophyll-a/b protein 2	0.086	2.1	7.220 × 10^6^	3.412 × 10^6^	down
1255	Heat shock protein HSP70	0.021	2.9	3.357 × 10^5^	9.837 × 10^5^	up

**Table 5:** Mass spectrometry (MS) identification of spots.

Class	Spot Number	MW [kDa]/pI Theoretical	Score	SC [%]	Peptides	Hypothetical Function	Protein Accession Number
Carbon and energy metabolism	3056	64.2/4.6	130.4	4.2	4	Carbonic anhydrase	P54212
	1209	54.5/5.2	62.4	4.8	2	ATP synthase CF1 *alpha* subunit (chloroplast)	ACS95056
	2218	54.5/5.2	100.7	5.0	2	ATP synthase CF1 *alpha* subunit (chloroplast)	ACS95056
	1187	51.8/4.8	3017.7	89.6	82	ATP synthase CF1 *beta* subunit (chloroplast)	ACS95063
	1246	51.8/4.8	71.7	9.8	2	ATP synthase CF1 *beta* subunit (chloroplast)	ACS95063
	1191	51.8/4.8	754.6	40.6	13	ATP synthase CF1 *beta* subunit (chloroplast)	ACS95063
	2259	28.2/9	67.9	8.3	2	Chloroplast ribulose phosphate-3-epimerase	AEF79975
Carbohydrate metabolism (Glycolysis/Gluconeogenesis)	1144	52.1/5.3	2202.8	55.5	51	Enolase	AIJ00881
Protein chaperon (stress)	1255	71.7/5.2	459.2	11.8	13	Heat shock protein HSP70	CAB71138
	1246	71.7/5.2	217.6	8.2	5	Heat shock protein HSP70	CAB71138
Photosynthesis	2391	24.7/6.4	200.0	15.4	5	Photosystem I light-harvesting chlorophyll-a/b protein	ACN94453
	2346	24.7/6.4	161.9	16.7	4	harvesting chlorophyll-a/b protein 2	ACN94453
Cellular Processes (Cytoskeleton)	1187	39.1/4.7	952.7	46.1	22	beta-tubulin, partial	AAY84712
	1187	49.4/4.9	326.5	19.7	9	alpha-tubulin protein	AEF79970
	1191	39.1/4.7	1683.3	57.3	47	beta-tubulin, partial	AAY84712
	1191	49.4/4.9	489.9	26.6	13	alpha-tubulin protein	AEF79970
